# Splice-Correction Strategies for Treatment of X-Linked Agammaglobulinemia

**DOI:** 10.1007/s11882-014-0510-0

**Published:** 2015-02-01

**Authors:** Burcu Bestas, Janne J. Turunen, K. Emelie M. Blomberg, Qing Wang, Robert Månsson, Samir EL Andaloussi, Anna Berglöf, C. I. Edvard Smith

**Affiliations:** 1 Department of Laboratory Medicine, Clinical Research Center, Karolinska Institutet, Karolinska University Hospital, Novum Hälsovägen 7, 141 57 Huddinge, Sweden; 2 Department of Laboratory Medicine, Center for Hematology and Regenerative Medicine, Karolinska Institutet, Karolinska University Hospital, Huddinge, Sweden; 3 Department of Physiology, Anatomy and Genetics, University of Oxford, Oxford, UK

**Keywords:** Morpholino, Locked nucleic acid (LNA), Pre-mRNA splicing, Splice switching, Gene therapy, B cell, Mouse model

## Abstract

X-linked agammaglobulinemia (XLA) is a primary immunodeficiency disease caused by mutations in the gene coding for Bruton’s tyrosine kinase (BTK). Deficiency of BTK leads to a developmental block in B cell differentiation; hence, the patients essentially lack antibody-producing plasma cells and are susceptible to various infections. A substantial portion of the mutations in *BTK* results in splicing defects, consequently preventing the formation of protein-coding mRNA. Antisense oligonucleotides (ASOs) are therapeutic compounds that have the ability to modulate pre-mRNA splicing and alter gene expression. The potential of ASOs has been exploited for a few severe diseases, both in pre-clinical and clinical studies. Recently, advances have also been made in using ASOs as a personalized therapy for XLA. Splice-correction of *BTK* has been shown to be feasible for different mutations in vitro, and a recent proof-of-concept study demonstrated the feasibility of correcting splicing and restoring BTK both ex vivo and in vivo in a humanized bacterial artificial chromosome (BAC)-transgenic mouse model. This review summarizes the advances in splice correction, as a personalized medicine for XLA, and outlines the promises and challenges of using this technology as a curative long-term treatment option.

## Introduction

Agammaglobulinemias are B cell deficiencies caused by mutations in the genes that encode components of the B cell receptor (BCR) or its precursor (pre-BCR). X-linked agammaglobulinemia (XLA), also known as Bruton’s agammaglobulinemia, accounts for 85 % of the patients in this group and is characterized by low or negligible levels of immunoglobulins [1]. XLA was first described in 1952 [2] and is often considered the prototype of an immunodeficiency disease, since it was the first defect, where a defined immunological phenotype (lack of gammaglobulin) was identified. In the early 1990s, it was demonstrated that the disease was caused by the inactivation of Bruton’s tyrosine kinase (BTK) [3–5]. Mutations in the *BTK* gene result in a developmental block in the bone marrow at the stage where the transition between pro-B and pre-B cells takes place. In XLA, precursor B cells are present, but they fail to differentiate [6, 7]. Hence, the amount of peripheral B cells is low, and they are of an immature phenotype [8], resulting in the absence of antigen-specific Ig production [9]. Female carriers are healthy, as the B lymphocytes with the X chromosome expressing the wild-type BTK are specifically selected for; in fact, only a single female with XLA has been definitively reported [10].

Mouse models have been extensively used to study the mechanisms of the immunodeficiency, and dysfunction of the mouse Btk was also identified as the underlying defect in mice affected by X-linked immunodeficiency (XID) [11–13]. This was subsequently confirmed by mouse models with engineered *Btk* knockouts (KO), which have essentially the same phenotype as the XID mice [14–16]. These mice have a 50 % reduction in the number of splenic B cells and reduced levels of secretory IgM and IgG3 and impaired responses to certain T cell-independent antigens [17]. In humans, the point mutation found in XID mice causes classical XLA; Btk deficiency therefore results in a less severe phenotype in mice [18].

XLA patients are vulnerable to bacterial and enteroviral infections. Encapsulated bacteria such as *Haemophilus influenza* and *Streptococcus pneumonia* are the most typical causes of bacterial infections [19–23]. Clinically, XLA patients display infections in the upper and lower respiratory and gastrointestinal tract [24]. Currently, there is no curative therapy for XLA, and the treatment instead consists of immunoglobulin substitution and frequent administration of antibiotics. This is suboptimal [25], since the patients’ quality of life is reduced owing to recurrent infections [20, 26, 23, 24]. Some attempts have been made to treat XLA patients by stem cell transplantation, but the results have not been satisfactory due to transplantation complications [27]. Therefore, alternative strategies such as gene therapy remain valid [1, 17]. In this review, we will briefly discuss one such putative XLA therapy, *BTK* splice-correction, and its possible future applications.

## BTK Belongs to a Family of Kinases and Signals Downstream of the B Cell Receptor

BTK is expressed from a 37.5-kb gene that contains 19 exons and has a molecular weight of 77 kDa [28–31]. It belongs to the TEC family of non-receptor kinases (TFKs), consisting of additional four members: TEC, BMX, ITK, and TXK/RLK [32]. Among those, BTK and ITK are the only members definitively associated with human disease [33]. While BTK deficiency causes XLA, mutations inactivating ITK result instead in susceptibility to severe Epstein-Barr virus infections (reviewed in [34]). ITK is also involved in the formation of a fusion gene causing T cell lymphomas [35–38]. BTK is expressed in myeloid cells and in B lineage cells with the important exception of mature plasma cells [39–41]. Although the phenotypic alterations caused by *BTK* mutations are predominantly limited to the B cell lineage, there have been reports of other affected cell lineages as well [42, 43].

Similar to other TFKs, BTK has unique domains that are important for downstream signaling [32]. These are from the N terminus: pleckstrin homology (PH), Tec homology (TH), Src homology 3 (SH3), SH2, and the catalytic kinase domain [42]. Upon BCR stimulation, BTK translocates to the plasma membrane, where it is phosphorylated at Y551 of the kinase domain by SRC family kinases. Following the *trans*-phosphorylation, BTK becomes auto-phosphorylated at Y223 in its SH3 domain [44, 45]. BTK subsequently phosphorylates phospholipase Cγ2 (PLCγ2), which leads to the activation of downstream effectors such as NF-κB and increased calcium flux. These events are important for the maturation, survival, and proliferation [42, 46] of B cells, and BTK-deficient B cells lack the NF-κB auto-regulatory network [47–49].

## Mutations in the *BTK* Gene

**Fig. 1:**
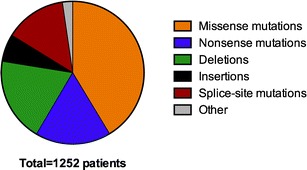
Distribution of the most common type of *BTK* mutations using an updated version of BTKbase [54]

More than 800 mutations have been described for *BTK*. Many de novo mutations have been reported and no single alteration accounts for more than 3–6 % of all mutations. Genetic changes causing XLA have been identified throughout the *BTK* gene with the exception of the SH3 domain, where no missense mutations have been reported [3, 5, 18, 29, 46, 50, 51]. Common mutations include amino acid substitutions, premature stop codons, splice site defects, and frameshifts caused by deletions or insertions. Some mutations, such as amino acid substitutions in non-invariant sites, and splicing defects, can result in the expression of low levels of (partially active) BTK, causing a milder form of XLA [52, 53, 50]. The information regarding all known mutations in XLA patients has been collected and is available in the BTKbase database (http://structure.bmc.lu.se/idbase/BTKbase/) [54]. Figure 1 shows the frequency of different types of *BTK* mutations from 1252 patients as presented in the BTKbase.

## *BTK* Splice Site Mutations in XLA

**Fig. 2:**
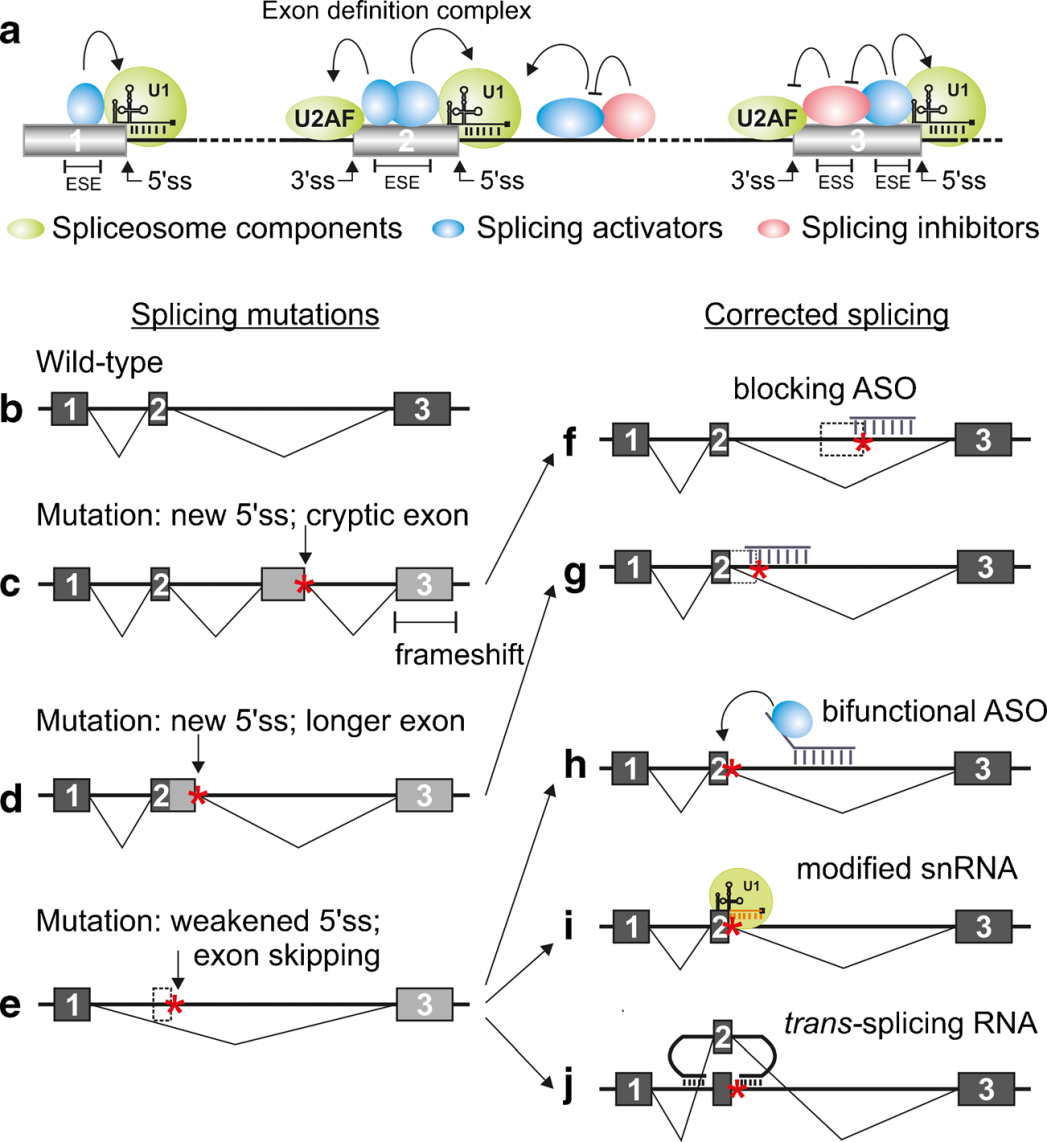
Splicing mechanism and splice correction methods. **a** Splice sites are initially recognized by spliceosome components binding to the ends of the exons (exon definition). The recognition is further regulated by activating or inhibitory splicing factors. Later, the factors within the same intron form complexes that excise the intron from the mRNA (not shown). Different factors are represented using a color code, as indicated. **b**–**j** Effect of mutations on splicing and possible splice correction methods. On the left side of the panel, splicing of a wild-type pre-mRNA is depicted schematically (**b**), followed by mutants with inclusion of a cryptic exon (**c**), activation of an alternative, cryptic splice site (**d**), and exclusion of a coding exon (**e**). On the right side of the panel, different correction methods are depicted for each mutant type, as indicated by *arrows*: Blocking the cryptic splice sites with ASOs restores correct splicing (**f**, **g**). Bifunctional ASO recruits splicing factors to enhance exon recognition (**h**). Exon inclusion is restored by a U1 snRNA, which has been modified to better recognize the mutated splice site (**i**). Trans-splicing RNA with the correct splice sites replaces the faulty exon and the splicing machinery recognizes it as the wild-type exon (**j**). *ASO* antisense oligonucleotide, *ESE*/*ESS* exonic splicing enhancer/silencer sequence

A sizeable group of XLA mutations cause splicing defects in the *BTK* pre-mRNA. As in most human genes, the open reading frame is not contiguous but is composed of separate exons disrupted by intronic sequences. These introns must be removed in a precise manner before an mRNA is ready to be used as template for protein synthesis. This process is carried out by the spliceosome, a macromolecular machinery, which is composed of a number of small nuclear RNAs (snRNAs) and associated protein complexes [55]. In addition to being precise, the splicing process also has to be flexible, since over 92 % of all human pre-mRNA transcripts are spliced alternatively to produce different mRNA and protein isoforms from the same gene [56], often in response to external stimuli. To achieve this regulation, the spliceosome employs a number of accessory proteins that can activate, or inhibit, the recognition of splice sites, such that the combined effect of inhibitors and activators effectively decides whether a given splice site is used (Fig. 2a). This flexibility also poses risks, since it can result in aberrantly spliced transcripts when the pre-mRNA contains mutations, as in many XLA cases.

**Fig. 3:**
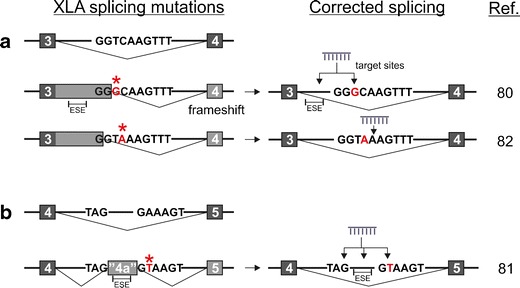
Mechanism of splice correction by ASOs for XLA. **a** Mutation in the intronic region causes the activation of an aberrant splice site, resulting in an extended exon. ASOs are used to block the exonic splicing enhancer (ESE) regions or the aberrant splice site in order to restore correct pre-mRNA splicing. **b** Intronic mutation activates a cryptic splice site and introduces a cryptic exon. ASOs blocking the ESEs or the splice sites restore the correct splicing pattern. The mutated nucleotide is highlighted in *red*, and also with an *asterisk*. *ASO* antisense oligonucleotide

As reported by a large number of studies [57, 52, 58–64], splice site mutations comprise a substantial portion of all mutations causing XLA. These mutations are spread throughout the gene, and frequently consist of single nucleotide changes, but may also be caused by insertions or deletions of larger sequence elements. The mutations occur not only in the invariant splice-donor sites or splice-acceptor sites but also in other intronic or exonic locations. The mechanisms by which BTK expression is affected also differ. A common type of mutation disrupts splice sites or weakens enhancer sequences, which typically results in the exclusion of the whole exon from the mRNA, even when the mutation only affects one splice site directly (Fig. 2e). This is due to the fact that, in the initial phases of the spliceosome assembly, the splice sites are generally recognized as pairs over the exons (“exon definition”), aided by activator proteins that link together the splicing factors located at the ends of the exon (Fig. 2a) [55]. Conversely, an intronic mutation can create a novel (cryptic) splice site, causing the inclusion of a cryptic exon (Fig. 2c), due to exon definition interactions with a splice site-like sequence already present in the pre-mRNA (see, e.g., Fig. 3b). However, both types of mutations can also result in the activation of alternative splice sites, leading to the inclusion of intronic sequences as extensions to the coding exons (Fig. 2d), or exclusions of a portion of an exon. These changes generally lead to disruption of the reading frame, or possibly in a shortened, or lengthened, non-functional BTK protein. Such defective proteins are expected to be rapidly degraded [50]. Correcting the splicing defects could therefore rescue the expression of the functional, endogenous BTK protein. These particular mutations hence form an interesting target group for personalized RNA therapeutics and are explored in the following sections.

## Splice Correction Strategies

The strategy required to correct splicing defects depends on the nature of the mutation in question and is therefore a prime example of personalized medicine. The correction is most straightforward for defects caused by the activation of a cryptic splice site (Fig. 2c). Here, splice correction can be achieved by sterically blocking the access of splicing factors to either the cryptic splice sites themselves or nearby splicing enhancer sequences (Fig. 2f, g). In the simplest form, this can be done by administering short (15–25 nt) antisense oligonucleotides (ASOs). A number of splicing defects have been corrected with this methodology using in vivo models [65, 66], including hematopoietic cell defects such as beta-thalassemia [67]. An advanced example of this type of therapy are the ASOs used to correct the reading frame of the *DMD* mRNA in Duchenne muscular dystrophy, with clinical studies in phases II/III completed [68].

The other main type of splicing defect, caused by the exclusion of *bona fide* exons (Fig. 2e), is a somewhat more challenging scenario. In some cases, it is possible to find inhibitory sites near the affected exons that can be blocked with simple ASOs, thus relieving the inhibition and allowing more efficient splicing (e.g., in the treatment of spinal muscular atrophy (SMA) [69]). However, this may not be the case for all exons due to lack of such active inhibition. In those cases, it will be necessary to actively recruit splicing factors that promote splicing. One way is to use bi-functional oligonucleotides that have an antisense sequence targeting them to the desired site, as well as an unpaired “tail” sequence, that is able to bind splicing activator proteins (Fig. 2h). Currently, this method remains largely unexplored, although some advances have been made, e.g., in in vitro SMA models [70].

Another method for enhancing inclusion of skipped exons is to use modified components of the spliceosome itself. This can be achieved, for example, by modifying the sequence of the U1 snRNA, which is the factor involved in recognizing the 5′ splice site (5′ss) of introns, so that its antisense sequence is a better match to the mutated splice site (Fig. 2i). An even more exciting method is to use the spliceosome’s capacity to catalyze splicing between two RNA molecules in what is known as *trans*-splicing. In this method, an RNA molecule with the corrected exon sequence is targeted to the affected site and is then spliced into the maturing mRNA (Fig. 2j). This approach has the added benefit that it could in principle be used to correct not only those defects that are caused by splicing errors but also those caused by missense mutations or even (small) deletions. However, both the snRNAs and *trans*-splicing RNAs must be transcribed from expression vectors and therefore require advanced delivery systems. For example, viral vectors have been used for correction of coagulation factor deficiency by modified snRNA and *trans*-splicing in mouse models [71, 72]. For the purposes of this review, we will concentrate on the function of the simpler ASO agents. Readers interested in the possibilities of using (*trans*-) RNA-based correction for XLA are advised to turn to an earlier recent review of ours [1].

In addition to selecting the optimal RNA target site and mode of action, the ASO also has to be enhanced for optimal specificity, stability, and delivery to the target tissues and cells. The fact that ASOs are chemically synthesized allows for wide range of chemical modifications that can achieve these goals. These modifications generally appear either in the sugar moiety or in the backbone. The most commonly used modifications are 2′-*O*-methyl phosphorothioate (2′OMePS), phosphorodiamidate morpholino oligomer (PMO), locked nucleic acid (LNA), and 2′-methoxyethoxy (MOE). All of these modifications provide increased nuclease stability, and most of them also change the affinity of the ASO for its target RNA, with LNA modification giving the strongest increase in binding affinity [73]. On the flipside, the increased affinity of LNA may compromise target specificity, and careful optimization of the specificity is thus required when designing the ASO [74]. In contrast to the other chemistries, PMO oligomers are charge neutral and are therefore not able to bind to serum proteins in vivo. This property makes them potentially less potent at lower doses due to extensive renal clearance [75]. However, PMOs are extremely well tolerated in vivo and even repeated doses as high as 1.5 g/kg appear to have negligible side effects in mice [76]. In order to increase the potency of PMO oligomers, they have been conjugated to cell-penetrating peptides (CPPs), which have the ability to rapidly translocate across cellular membranes, thereby enhancing cellular uptake. Different ASO chemistries and CPPs are reviewed in [77–79].

## Recent Advances in Applying Splice Correction Therapies to XLA Models

While splice correction is a relatively novel therapeutic strategy for XLA, three recent studies by us and others have shown the feasibility of using ASOs for correcting BTK expression [80•, 81••, 82•]. In each of these cases, the cause was found to be an intronic mutation creating a new splice site, namely the inclusion of a cryptic exon in intron 4 (exon “4a”) (Fig. 3b) [81••], or activation of new alternative 5′ splice sites (5′ss) in intron 3, leading to a lengthened exon 3 [80•, 82•] (Fig. 3a). In the latter case, the two publications described mutations at almost the exact same site (T to G at 5′ss + 108, or C to A at 5′ss + 109), both of which resulted in a similar activation of a cryptic 5′ss. Although the original 5′ss remains intact, preferential usage of the cryptic sites was observed in both cases. Interestingly, the T + 108G mutation creates a de novo GC-type 5′ss that are normally present at low frequency (1/100) in authentic introns [80•]. On further investigation, the cause behind this preferential activation was revealed to be the presence of an upstream exonic splicing enhancer (ESE) [80•]. The presence of ESEs was also inferred in our most recent publication, highlighting the importance of auxiliary elements for the activation of cryptic splice sites. Furthermore, the two studies show that blocking enhancer sequences by ASOs was at least as effective, or more effective, than blocking the core splice sites [81••, 80•].

The fact that enhancer sequences are also effective targets increases the available space for optimal ASO design. The selection of a correct target site is important not only for ASO efficacy but also for the specificity, in order to avoid binding to off-target sites in other transcripts. Indeed, it was found that a PMO ASO was able to block the cryptic splice site and correct *BTK* splicing efficiently in primary XLA PBMCs, even when the PMO had five mismatched positions [82•]. Thus, while this demonstrates the robustness of splice correction in vitro in patient cells, it may lead to deleterious off-target effects in vivo, making it crucial to optimize the specificity.

To be able to provide a proof of concept for *BTK* splice correction in vivo, we turned to mouse models. To our knowledge, there is only one existing transgenic animal model maintaining a hematopoietic disease caused by aberrant splicing [83]. In order to more specifically study the feasibility of using ASOs for human BTK restoration, we generated a transgenic mouse by using a bacterial artificial chromosome (BAC) construct carrying the human *BTK* gene with the intron 4 mutation causing XLA. A transgenic mouse was generated by pronuclear injection, followed by breeding onto a *Btk* KO strain. As a consequence, our model only has the mutated human *BTK* and no endogenous mouse *Btk*. Moreover, the fact that the BAC construct includes some human genes flanking *BTK*, including the splicing factor gene *HNRNPH2*, adds an additional human component to our model. Thus, as the only existing model of its kind, ours is of broad general interest for studying the treatment strategies for aberrant splicing in the lymphoid compartment.

The initial phase of the study included the optimization of the ASOs in vitro. Bioinformatic data about the presence of putative nearby ESEs was considered for the ASO design. The most successful ASOs were subsequently tested in the human transgenic mouse model, initially ex vivo. Since this mouse model has an XID phenotype, and therefore relatively high levels of splenic B cells, these cells were isolated for ASO treatment. We were able to restore BTK mRNA and protein expression by electroporation of LNA-modified ASOs or by using cell-penetrating peptide conjugated PMO (CPP-PMO) both in the mouse primary splenic B cells and in XLA patient monocytes. We further analyzed the functionality of the restored protein by assessing cell survival and *trans*-phosphorylation of BTK. Stimulated primary mouse B cells from the BAC-transgenic mouse showed phosphorylation of BTK at Y551 and enhanced survival after ASO treatment, indicating that BTK was catalytically active and functional.

Importantly, the ex vivo treatment was also able to rescue BTK in differentiated pro-B cells from the bone marrow (BM) of the BAC-transgenic mouse. Since XLA patients have the developmental block at the pro-B cell stage, the ability to target this cell population is an important indication of the clinical relevance of the splice correction treatment strategy. As a proof of concept for in vivo delivery of the splice-correcting ASOs, a systemic injection to the BAC-transgenic mouse with the CPP-PMO conjugates was performed. Very encouragingly, after 1 week, BTK was restored in B cells isolated from the spleen and bone marrow of the in vivo-treated mice. Taken together, these results demonstrate the feasibility of correcting BTK defects by ASOs both ex vivo and in vivo, paving the way for new treatment options for XLA.

## Germinal Centers Are Crucial for the Generation of Pathogen-Specific Plasma Cells

The developmental defect in XLA manifests itself in immature B cell progenitors, and the treatment therefore needs to primarily target the defective pro-B cells. In the bone marrow, proliferating precursor B cells are located mainly in the periphery, close to the surrounding bone, and intimately associated with stromal reticular cells [84]. Once the B cell progenitors differentiate and reach a more developed stage, they leave the bone marrow and enter peripheral lymphoid tissues such as lymph nodes and spleen to undergo maturation in germinal centers (GCs). More specifically, GCs are the sites for B cell clonal amplification and somatic hypermutation (SHM), yielding high affinity antibodies [85]. The GCs attract naïve B cells, which make up primary follicles in the spleen and lymph nodes before exposure to antigen. The developing GC is divided into two distinct compartments, one of which is the light zone (LZ), where selection of high affinity B cells take place. This selection is followed by proliferation and somatic hypermutation in the dark zone (DZ) and subsequently another round of affinity selection in the LZ [86]. A crucial component for the GC B cells is the transcription factor Bcl-6, which regulates and directs their phenotype [87]. This process is controlled by T follicular helper (Tfh) cells [88] that form a unique T cell subset, which is also regulated by Bcl-6.

GC B cells may either undergo apoptosis or differentiate into plasma cells or memory B cells. Plasma cells are committed to antibody production and they also have the ability to perform class-switching during maturation [89]. In contrast, memory B cells form a dormant population are capable of responding faster once they encounter a former pathogen. Memory B cells are generated along two unique differentiation pathways, one GC dependent and the other GC independent. The former cells are somatically mutated and express high-affinity antibodies, while the latter are not mutated, expressing low-affinity antibodies [90].

From a therapeutical point of view, the restoration of BTK alone cannot reconstitute the immune system of the XLA patient. Generation of plasma cells and memory B cells are the key events for raising an immune response. Since the ASO therapy provides a transient restoration, additional strategies supporting the maturation and survival of antibody-producing B cells are required, such as immunization. Thus, peripheral lymphoid tissues are the most crucial sites for a supportive therapy, which will be explored in the following section.

## The Treatment Strategy for Splice Correction in XLA and Future Prospects

The rationale for the splice-correcting treatment strategy in XLA is to first overcome the differentiation block and generate mature B cells. The subsequent step is to immunize patients with the microbes that XLA patients are susceptible to, with the aim of inducing the formation of long-lived plasma cells. While plasma cells are end-stage B lineage cells, they differ considerably from all the earlier stages. Of particular importance for our treatment concept is the fact that plasma cells are not dependent on BTK expression. Since plasma cells can live for several years, a transient treatment of the splicing defect could ideally generate a long-term immunoprotective effect. Therefore, the treatment strategy for splice correction in XLA includes two phases: restoration of BTK followed by immunization that generates a protective humoral immune response.

**Fig. 4:**
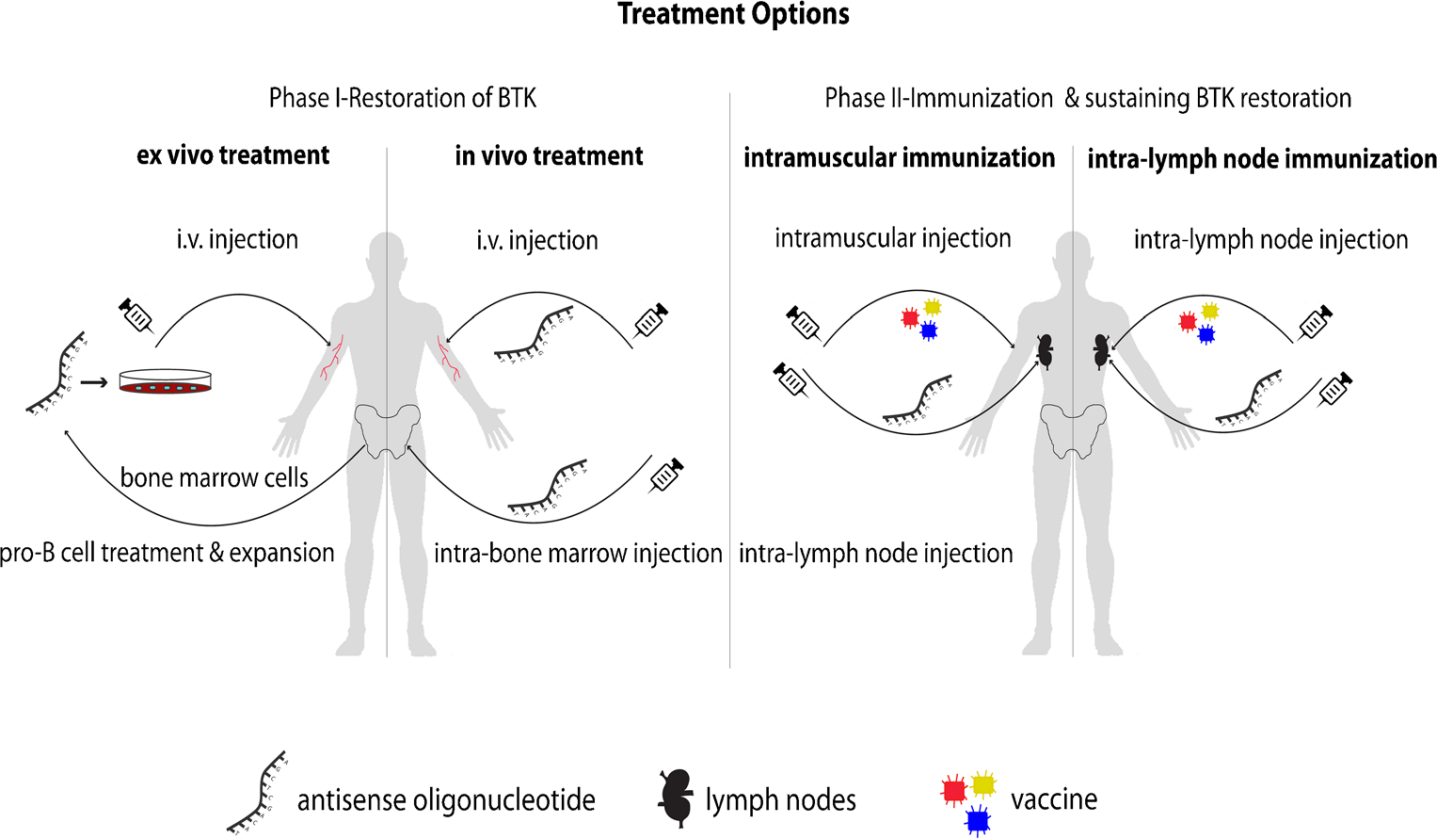
Proposed treatment options for XLA. The treatment strategy involves two phases. Phase I is the restoration of BTK expression. In the ex vivo part, bone marrow cells are collected from the patient. After the ASO treatment, they are re-injected intravenously (i.v.). In the in vivo part, ASOs are injected either i.v. or directly into the bone marrow. Phase II consists of the immunization during sustained expression of BTK. One possibility is to perform intramuscular vaccination combined with ASO injection to the lymph node. The other option is to inject both the ASO and the vaccine into the lymph node. *ASO* antisense oligonucleotide

Based on our experiments in transgenic mice carrying the mutated human *BTK* gene, we know that B cell progenitors can be treated ex vivo, thereby restoring BTK protein expression. Hence, one possibility is to correct pro-B cells ex vivo and re-infuse them into the patient, where they subsequently home to the lymphoid tissues (Fig. 4—phase I). Homing of the injected pro-B cells into the bone marrow of mice has been reported previously [91]. Moreover, intravenous injection of stem cells to cancer patients for reconstituting the immune system is used in the clinic, and the injected cells are known to localize to the bone marrow of the patient. An alternative method to the ex vivo treatment would be the systemic infusion of ASOs into the patient (Fig. 4—phase I). We have demonstrated in our experimental model that this is feasible, i.e., systemic administration of ASOs can restore the expression of BTK both in the bone marrow and in the spleen of BTK-defective mice [81••].

Owing to the fact that an infusion would require rather large amounts of ASOs, an alternative strategy would be to deliver the ASOs directly into the bone marrow, e.g., into the back of the pelvic bone (Fig. 4—phase I). This form of delivery would lower the cost and could also reduce off-target effects caused by the uptake into cells outside the bone marrow. While these treatments would restore BTK expression in the progenitor cells, the duration of the expression is crucial, since the more mature B lineage cells would leave the bone marrow to home to the secondary lymphoid organs. Thus, at this point, a secondary phase treatment strategy is needed to support these cells that would still be dependent on *BTK* splice correction for their survival.

The aim of the second phase of the treatment would be to develop a potent humoral immunity against those infectious agents to which XLA patients are susceptible (Fig. 4—Phase II). This will be achieved by immunization using commercially available vaccines. During vaccination, BTK expression is required for the survival of the maturing B cells in the peripheral lymphoid tissues. The challenge is to reduce the required amount of ASOs as much as possible. In one scenario, ASOs would preferentially be delivered locally into the secondary lymphoid organ, where the immune response takes place.

Let us first consider the immunization. Vaccines are mostly administered subcutaneously or intramuscularly. However, given the aim to pursue local administration of ASOs for this patient category, immunization directly into the lymph nodes might be a better option, since this is where the immune-competent cells normally reside. To this end, intra-lymph node injections have been reported to result in more specific immune responses compared to other administration routes for the immunotherapy of cancer patients, as well as in allergy [92–94]. Intra-lymph node injection of antigens has also been tried in mice [95] with the aim of injecting a biodegradable vaccine formulation to the lymph nodes through a non-surgical method [96]. Such delivery vehicles with a controlled release of antigens into the lymph nodes might thus represent a future option in the clinic. Moreover, recent evidence indicates that assistance from Tfhs is a limiting factor for vaccine responses in humans as reviewed in [88]. This also suggests that manipulation of Tfh cells potentially could boost vaccine responses, e.g., by reducing the effect of inhibitory signaling pathways, and such a therapeutic blockade has recently been reported [97].

In view of these developments, for sustained BTK expression, ASOs could potentially either be administered into regions that are drained to a particular lymph node or, alternatively, be injected directly into the lymph node. The local delivery may also be of particular importance in XLA, since the secondary lymphoid organs are not fully developed in these patients. This could perhaps also mean that the immunization may take more time than in healthy individuals, further increasing the amount of ASOs needed for BTK restoration during the vaccination. Collectively, the suggested complementing treatment strategy should theoretically allow the maturation of B lymphocytes into plasma cells, generating long-term immune protection.

In our experience, B cells are relatively hard to transfect with ASOs. Others have also noted the difficulty of delivering MOE and LNA-modified ASOs to the erythroid progenitor cells, which are of hematopoietic origin like the B cells [67]. Furthermore, for splice correction, the ASOs also need to be transported to the nucleus of the cells. For these reasons, obtaining a meaningful effect would generally require a high concentration of the ASO. In our hands, most ASO derivatives were only efficiently taken up by the B cells using electroporation ex vivo. However, since this is a rather harsh method for the cells, the treatments could likely be improved by alternative delivery methods. Very promisingly, the PMO oligomers conjugated with CPPs gave satisfactory BTK restoration both ex vivo and in vivo. PMO oligomers with or without CPP conjugates have also been shown by others to be taken up by hematopoietic cells both ex vivo and in vivo [67, 98, 99], suggesting that further development of PMO oligomers with different CPP conjugates is a promising platform for future studies. Likewise, further development of other ASO derivatives that are more readily taken up by the B cells may also provide alternatives for more effective treatments. In this regard, ASOs with mixed LNA/2′OMePS content remain strong candidates for this development due to their high target affinity.

For the purpose of pre-clinical investigation of ASOs, a mouse model has its own advantages and disadvantages. Regarding the phenotype, our BAC-transgenic model has only a partial differentiation block as compared to humans, where essentially no functional B cells are generated. Removing another *BTK*-related gene, *TEC*, enhances the disease severity [100]. Thus, a transgenic mouse model on a BTK/TEC double KO background is a better disease model for the analysis of phenotypic changes after the restoration of BTK. Moreover, to be able to translate the results to the clinics, one might need to experiment on other animal models, since pharmacokinetic or pharmacodynamic properties of the ASOs differ between species [101]; for example, it is known that mice tolerate CPPs at higher doses than rats [102].

## Conclusion

As for many other genetic diseases, gene therapy represents a future option for XLA. The transient need of BTK in B cells, which differentiate into long-lived protective plasma cells, makes splice correction therapy particularly feasible for XLA. With our recent report, we have shown the proof of concept for this type of therapy in XLA. In order to have a long-term curative effect, BTK restoration needs to be combined with supplementary vaccination strategy. To this end, optimizing the delivery method for minimal amount of ASOs and defining the optimal duration for BTK restoration are crucial parameters to be considered. Thus, the possibility of targeting B cells and correcting the splicing defects opens up many options and could potentially be extended to the treatment of other hematopoietic diseases.

## References

[1] Berglöf A, Turunen JJ, Gissberg O, Bestas B, Blomberg KE, Smith CI (2013). Agammaglobulinemia: causative mutations and their implications for novel therapies. Expert Rev Clin Immunol.

[2] Bruton OC (1952). Agammaglobulinemia. Pediatrics.

[3] Vetrie D, Vorechovský I, Sideras P, Holland J, Davies A, Flinter F (1993). The gene involved in X-linked agammaglobulinaemia is a member of the src family of protein-tyrosine kinases. Nature.

[4] Tsukada S, Saffran DC, Rawlings DJ, Parolini O, Allen RC, Klisak I (1993). Deficient expression of a B cell cytoplasmic tyrosine kinase in human X-linked agammaglobulinemia. Cell.

[5] Vihinen M, Kwan SP, Lester T, Ochs HD, Resnick I, Väliaho J (1999). Mutations of the human BTK gene coding for Bruton tyrosine kinase in X-linked agammaglobulinemia. Hum Mutat.

[6] Nomura K, Kanegane H, Karasuyama H, Tsukada S, Agematsu K, Murakami G (2000). Genetic defect in human X-linked agammaglobulinemia impedes a maturational evolution of pro-B cells into a later stage of pre-B cells in the B-cell differentiation pathway. Blood.

[7] Noordzij JG, de Bruin-Versteeg S, Comans-Bitter WM, Hartwig NG, Hendriks RW, de Groot R (2002). Composition of precursor B-cell compartment in bone marrow from patients with X-linked agammaglobulinemia compared with healthy children. Pediatr Res.

[8] Conley ME (1985). B cells in patients with X-linked agammaglobulinemia. J Immunol.

[9] Sideras P, Smith CI (1995). Molecular and cellular aspects of X-linked agammaglobulinemia. Adv Immunol.

[10] Takada H, Kanegane H, Nomura A, Yamamoto K, Ihara K, Takahashi Y (2004). Female agammaglobulinemia due to the Bruton tyrosine kinase deficiency caused by extremely skewed X-chromosome inactivation. Blood.

[11] Thomas JD, Sideras P, Smith CI, Vorechovský I, Chapman V, Paul WE (1993). Colocalization of X-linked agammaglobulinemia and X-linked immunodeficiency genes. Science.

[12] Rawlings DJ, Saffran DC, Tsukada S, Largaespada DA, Grimaldi JC, Cohen L (1993). Mutation of unique region of Bruton’s tyrosine kinase in immunodeficient XID mice. Science.

[13] Wicker LS, Scher I (1986). X-linked immune deficiency (xid) of CBA/N mice. Curr Top Microbiol Immunol.

[14] Khan WN, Alt FW, Gerstein RM, Malynn BA, Larsson I, Rathbun G (1995). Defective B cell development and function in Btk-deficient mice. Immunity.

[15] Kerner JD, Appleby MW, Mohr RN, Chien S, Rawlings DJ, Maliszewski CR (1995). Impaired expansion of mouse B cell progenitors lacking Btk. Immunity.

[16] Hendriks RW, de Bruijn MF, Maas A, Dingjan GM, Karis A, Grosveld F (1996). Inactivation of Btk by insertion of lacZ reveals defects in B cell development only past the pre-B cell stage. EMBO J.

[17] Hendriks RW, Bredius RG, Pike-Overzet K, Staal FJ (2011). Biology and novel treatment options for XLA, the most common monogenetic immunodeficiency in man. Expert Opin Ther Targets.

[18] Vihinen M, Belohradsky BH, Haire RN, Holinski-Feder E, Kwan SP, Lappalainen I (1997). BTKbase, mutation database for X-linked agammaglobulinemia (XLA). Nucleic Acids Res.

[19] Ochs HD, Smith CI (1996). X-linked agammaglobulinemia. A clinical and molecular analysis. Medicine (Baltimore).

[20] Hermaszewski RA, Webster AD (1993). Primary hypogammaglobulinaemia: a survey of clinical manifestations and complications. Q J Med.

[21] Lederman HM, Winkelstein JA (1985). X-linked agammaglobulinemia: an analysis of 96 patients. Medicine (Baltimore).

[22] McKinney RE, Katz SL, Wilfert CM (1987). Chronic enteroviral meningoencephalitis in agammaglobulinemic patients. Rev Infect Dis.

[23] Plebani A, Soresina A, Rondelli R, Amato GM, Azzari C, Cardinale F (2002). Clinical, immunological, and molecular analysis in a large cohort of patients with X-linked agammaglobulinemia: an Italian multicenter study. Clin Immunol.

[24] Winkelstein JA, Marino MC, Lederman HM, Jones SM, Sullivan K, Burks AW (2006). X-linked agammaglobulinemia: report on a United States registry of 201 patients. Medicine (Baltimore).

[25] Cunningham-Rundles C (2011). Key aspects for successful immunoglobulin therapy of primary immunodeficiencies. Clin Exp Immunol.

[26] Gardulf A, Björvell H, Gustafson R, Hammarström L, Smith CI (1993). The life situations of patients with primary antibody deficiency untreated or treated with subcutaneous gammaglobulin infusions. Clin Exp Immunol.

[27] Howard V, Myers LA, Williams DA, Wheeler G, Turner EV, Cunningham JM (2003). Stem cell transplants for patients with X-linked agammaglobulinemia. Clin Immunol.

[28] Rohrer J, Parolini O, Belmont JW, Conley ME, Parolino O (1994). The genomic structure of human BTK, the defective gene in X-linked agammaglobulinemia. Immunogenetics.

[29] Ohta Y, Haire RN, Litman RT, Fu SM, Nelson RP, Kratz J (1994). Genomic organization and structure of Bruton agammaglobulinemia tyrosine kinase: localization of mutations associated with varied clinical presentations and course in X chromosome-linked agammaglobulinemia. Proc Natl Acad Sci U S A.

[30] Sideras P, Müller S, Shiels H, Jin H, Khan WN, Nilsson L (1994). Genomic organization of mouse and human Bruton’s agammaglobulinemia tyrosine kinase (Btk) loci. J Immunol.

[31] Hagemann TL, Chen Y, Rosen FS, Kwan SP (1994). Genomic organization of the Btk gene and exon scanning for mutations in patients with X-linked agammaglobulinemia. Hum Mol Genet.

[32] Smith CI, Islam TC, Mattsson PT, Mohamed AJ, Nore BF, Vihinen M (2001). The Tec family of cytoplasmic tyrosine kinases: mammalian Btk, Bmx, Itk, Tec, Txk and homologs in other species. BioEssays News Rev Mol Cell Dev Biol.

[33] Hussain A, Yu L, Faryal R, Mohammad DK, Mohamed AJ, Smith CI (2011). TEC family kinases in health and disease–loss-of-function of BTK and ITK and the gain-of-function fusions ITK-SYK and BTK-SYK. FEBS J.

[34] Ghosh S, Bienemann K, Boztug K, Borkhardt A (2014). Interleukin-2-inducible T-cell kinase (ITK) deficiency—clinical and molecular aspects. J Clin Immunol.

[35] Streubel B, Vinatzer U, Willheim M, Raderer M, Chott A (2006). Novel t(5;9)(q33;q22) fuses ITK to SYK in unspecified peripheral T-cell lymphoma. Leukemia.

[36] Hussain A, Mohammad DK, Gustafsson MO, Uslu M, Hamasy A, Nore BF (2013). Signaling of the ITK (interleukin 2-inducible T cell kinase)-SYK (spleen tyrosine kinase) fusion kinase is dependent on adapter SLP-76 and on the adapter function of the kinases SYK and ZAP70. J Biol Chem.

[37] Dierks C, Adrian F, Fisch P, Ma H, Maurer H, Herchenbach D (2010). The ITK-SYK fusion oncogene induces a T-cell lymphoproliferative disease in mice mimicking human disease. Cancer Res.

[38] Pechloff K, Holch J, Ferch U, Schweneker M, Brunner K, Kremer M (2010). The fusion kinase ITK-SYK mimics a T cell receptor signal and drives oncogenesis in conditional mouse models of peripheral T cell lymphoma. J Exp Med.

[39] de Weers M, Verschuren MC, Kraakman ME, Mensink RG, Schuurman RK, van Dongen JJ (1993). The Bruton’s tyrosine kinase gene is expressed throughout B cell differentiation, from early precursor B cell stages preceding immunoglobulin gene rearrangement up to mature B cell stages. Eur J Immunol.

[40] Smith CI, Baskin B, Humire-Greiff P, Zhou JN, Olsson PG, Maniar HS (1994). Expression of Bruton’s agammaglobulinemia tyrosine kinase gene, BTK, is selectively down-regulated in T lymphocytes and plasma cells. J Immunol.

[41] Nisitani S, Satterthwaite AB, Akashi K, Weissman IL, Witte ON, Wahl MI (2000). Posttranscriptional regulation of Bruton’s tyrosine kinase expression in antigen receptor-stimulated splenic B cells. Proc Natl Acad Sci U S A.

[42] Mohamed AJ, Yu L, Bäckesjö CM, Vargas L, Faryal R, Aints A (2009). Bruton’s tyrosine kinase (Btk): function, regulation, and transformation with special emphasis on the PH domain. Immunol Rev.

[43] López-Herrera G, Vargas-Hernández A, González-Serrano ME, Berrón-Ruiz L, Rodríguez-Alba JC, Espinosa-Rosales F (2014). Bruton’s tyrosine kinase—an integral protein of B cell development that also has an essential role in the innate immune system. J Leukoc Biol.

[44] Park H, Wahl MI, Afar DE, Turck CW, Rawlings DJ, Tam C (1996). Regulation of Btk function by a major autophosphorylation site within the SH3 domain. Immunity.

[45] Rawlings DJ, Scharenberg AM, Park H, Wahl MI, Lin S, Kato RM (1996). Activation of BTK by a phosphorylation mechanism initiated by SRC family kinases. Science.

[46] Conley ME, Broides A, Hernandez-Trujillo V, Howard V, Kanegane H, Miyawaki T (2005). Genetic analysis of patients with defects in early B-cell development. Immunol Rev.

[47] Bajpai UD, Zhang K, Teutsch M, Sen R, Wortis HH (2000). Bruton’s tyrosine kinase links the B cell receptor to nuclear factor κb activation. J Exp Med.

[48] Petro JB, Rahman SM, Ballard DW, Khan WN (2000). Bruton’s tyrosine kinase is required for activation of Iκb kinase and nuclear factor κb in response to B cell receptor engagement. J Exp Med.

[49] Yu L, Mohamed AJ, Simonson OE, Vargas L, Blomberg KE, Bjorkstrand B (2008). Proteasome-dependent autoregulation of Bruton tyrosine kinase (Btk) promoter via NF-κb. Blood.

[50] Lindvall JM, Blomberg KE, Väliaho J, Vargas L, Heinonen JE, Berglöf A (2005). Bruton’s tyrosine kinase: cell biology, sequence conservation, mutation spectrum, siRNA modifications, and expression profiling. Immunol Rev.

[51] Holinski-Feder E, Weiss M, Brandau O, Jedele KB, Nore B, Bäckesjö CM (1998). Mutation screening of the BTK gene in 56 families with X-linked agammaglobulinemia (XLA): 47 unique mutations without correlation to clinical course. Pediatrics.

[52] Noordzij JG, de Bruin-Versteeg S, Hartwig NG, Weemaes CM, Gerritsen EJ, Bernatowska E (2002). XLA patients with BTK splice-site mutations produce low levels of wild-type BTK transcripts. J Clin Immunol.

[53] Conley ME, Dobbs AK, Farmer DM, Kilic S, Paris K, Grigoriadou S (2009). Primary B cell immunodeficiencies: comparisons and contrasts. Annu Rev Immunol.

[54] Väliaho J, Smith CI, Vihinen M (2006). BTKbase: the mutation database for X-linked agammaglobulinemia. Hum Mutat.

[55] Will CL, Lührmann R. Spliceosome structure and function. Cold Spring Harb Perspect Biol. 2011;3(7). 10.1101/cshperspect.a003707 PMC3119917 21441581

[56] Wang Z, Burge CB (2008). Splicing regulation: from a parts list of regulatory elements to an integrated splicing code. RNA.

[57] Futatani T, Watanabe C, Baba Y, Tsukada S, Ochs HD (2001). Bruton’s tyrosine kinase is present in normal platelets and its absence identifies patients with X-linked agammaglobulinaemia and carrier females. Br J Haematol.

[58] Lopez-Herrera G, Berron-Ruiz L, Mogica-Martinez D, Espinosa-Rosales F, Santos-Argumedo L (2008). Characterization of Bruton’s tyrosine kinase mutations in Mexican patients with X-linked agammaglobulinemia. Mol Immunol.

[59] Fiorini M, Franceschini R, Soresina A, Schumacher RF, Ugazio AG, Rossi P (2004). BTK: 22 novel and 25 recurrent mutations in European patients with X-linked agammaglobulinemia. Hum Mutat.

[60] Wang Y, Kanegane H, Wang X, Han X, Zhang Q, Zhao S (2009). Mutation of the BTK gene and clinical feature of X-linked agammaglobulinemia in mainland China. J Clin Immunol.

[61] Tóth B, Volokha A, Mihas A, Pac M, Bernatowska E, Kondratenko I (2009). Genetic and demographic features of X-linked agammaglobulinemia in eastern and central Europe: a cohort study. Mol Immunol.

[62] Maekawa K, Yamada M, Okura Y, Sato Y, Yamada Y, Kawamura N (2010). X-linked agammaglobulinemia in a 10-year-old boy with a novel non-invariant splice-site mutation in Btk gene. Blood Cells Mol Dis.

[63] Zhang ZY, Zhao XD, Jiang LP, Liu EM, Wang M, Yu J (2010). Clinical characteristics and molecular analysis of 21 Chinese children with congenital agammaglobulinemia. Scand J Immunol.

[64] Qin X, Jiang LP, Tang XM, Wang M, Liu EM, Zhao XD (2013). Clinical features and mutation analysis of X-linked agammaglobulinemia in 20 Chinese patients. World J Pediatr.

[65] Hammond SM, Wood MJ (2011). Genetic therapies for RNA mis-splicing diseases. Trends Genet TIG.

[66] Havens MA, Duelli DM, Hastings ML (2013). Targeting RNA splicing for disease therapy. Wiley Interdiscip Rev RNA.

[67] Svasti S, Suwanmanee T, Fucharoen S, Moulton HM, Nelson MH, Maeda N (2009). RNA repair restores hemoglobin expression in IVS2-654 thalassemic mice. Proc Natl Acad Sci U S A.

[68] Hoffman EP, Connor EM (2013). Orphan drug development in muscular dystrophy: update on two large clinical trials of dystrophin rescue therapies. Discov Med.

[69] Douglas AG, Wood MJ (2013). Splicing therapy for neuromuscular disease. Mol Cell Neurosci.

[70] Owen N, Zhou H, Malygin AA, Sangha J, Smith LD, Muntoni F (2011). Design principles for bifunctional targeted oligonucleotide enhancers of splicing. Nucleic Acids Res.

[71] Balestra D, Faella A, Margaritis P, Cavallari N, Pagani F, Bernardi F (2014). An engineered U1 small nuclear RNA rescues splicing-defective coagulation F7 gene expression in mice. J Thromb Haemost JTH.

[72] Chao H, Mansfield SG, Bartel RC, Hiriyanna S, Mitchell LG, Garcia-Blanco MA (2003). Phenotype correction of hemophilia A mice by spliceosome-mediated RNA trans-splicing. Nat Med.

[73] Lundin KE, Højland T, Hansen BR, Persson R, Bramsen JB, Kjems J (2013). Biological activity and biotechnological aspects of locked nucleic acids. Adv Genet.

[74] Hammond SM, McClorey G, Nordin JZ, Godfrey C, Stenler S, Lennox KA (2014). Correlating in vitro splice switching activity with systemic in vivo delivery using novel ZEN-modified oligonucleotides. Mol Ther Nucleic Acids.

[75] Cirak S, Arechavala-Gomeza V, Guglieri M, Feng L, Torelli S, Anthony K (2011). Exon skipping and dystrophin restoration in patients with Duchenne muscular dystrophy after systemic phosphorodiamidate morpholino oligomer treatment: an open-label, phase 2, dose-escalation study. Lancet.

[76] Wu B, Xiao B, Cloer C, Shaban M, Sali A, Lu P (2011). One-year treatment of morpholino antisense oligomer improves skeletal and cardiac muscle functions in dystrophic mdx mice. Mol Ther J Am Soc Gene Therapy.

[77] Kole R, Krainer AR, Altman S (2012). RNA therapeutics: beyond RNA interference and antisense oligonucleotides. Nat Rev Drug Discov.

[78] El Andaloussi SA, Hammond SM, Mäger I, Wood MJ (2012). Use of cell-penetrating-peptides in oligonucleotide splice switching therapy. Curr Gene Ther.

[79] Copolovici DM, Langel K, Eriste E, Langel Ü (2014). Cell-penetrating peptides: design, synthesis, and applications. ACS Nano.

[80.•] Kralovicova J, Hwang G, Asplund AC, Churbanov A, Smith CI, Vorechovsky I (2011). Compensatory signals associated with the activation of human GC 5′ splice sites. Nucleic Acids Res.

[81.••] Bestas B, Moreno PM, Blomberg KE, Mohammad DK, Saleh AF, Sutlu T (2014). Splice-correcting oligonucleotides restore BTK function in X-linked agammaglobulinemia model. J Clin Invest.

[82.•] Rattanachartnarong N, Tongkobpetch S, Chatchatee P, Daengsuwan T, Ittiwut C, Suphapeetiporn K et al. In vitro correction of a novel splicing alteration in the BTK gene by using antisense morpholino oligonucleotides. Arch Immunol Ther Exp (Warsz). 2014. *The study shows the restoration of BTK splicing by PMO-based ASOs in peripheral blood mononuclear cells of an XLA patient*. 10.1007/s00005-014-0283-0 24658450

[83] Lewis J, Yang B, Kim R, Sierakowska H, Kole R, Smithies O (1998). A common human β-globin splicing mutation modeled in mice. Blood.

[84] Lu L, Osmond DG (2000). Apoptosis and its modulation during B lymphopoiesis in mouse bone marrow. Immunol Rev.

[85] Victora GD, Dominguez-Sola D, Holmes AB, Deroubaix S, Dalla-Favera R, Nussenzweig MC (2012). Identification of human germinal center light and dark zone cells and their relationship to human B-cell lymphomas. Blood.

[86] MacLennan IC (1994). Germinal centers. Annu Rev Immunol.

[87] Basso K, Saito M, Sumazin P, Margolin AA, Wang K, Lim WK (2010). Integrated biochemical and computational approach identifies BCL6 direct target genes controlling multiple pathways in normal germinal center B cells. Blood.

[88] Crotty S (2014). T follicular helper cell differentiation, function, and roles in disease. Immunity.

[89] Shapiro-Shelef M, Calame K (2005). Regulation of plasma-cell development. Nat Rev Immunol.

[90] Takemori T, Kaji T, Takahashi Y, Shimoda M, Rajewsky K (2014). Generation of memory B cells inside and outside germinal centers. Eur J Immunol.

[91] Rolink AG, Nutt SL, Melchers F, Busslinger M (1999). Long-term in vivo reconstitution of T-cell development by Pax5-deficient B-cell progenitors. Nature.

[92] Ribas A, Weber JS, Chmielowski B, Comin-Anduix B, Lu D, Douek M (2011). Intra-lymph node prime-boost vaccination against Melan A and tyrosinase for the treatment of metastatic melanoma: results of a phase 1 clinical trial. Clin Cancer Res Off J Am Assoc Cancer Res.

[93] Smith KA, Qiu Z, Wong R, Tam VL, Tam BL, Joea DK (2011). Multivalent immunity targeting tumor-associated antigens by intra-lymph node DNA-prime, peptide-boost vaccination. Cancer Gene Ther.

[94] Zaleska A, Eiwegger T, Soyer O, van de Veen W, Rhyner C, Soyka MB (2014). Immune regulation by intralymphatic immunotherapy with modular allergen translocation MAT vaccine. Allergy.

[95] Johansen P, Kündig TM (2014). Intralymphatic immunotherapy and vaccination in mice. J Visualized Exp JoVE.

[96] Andorko JI, Tostanoski LH, Solano E, Mukhamedova M, Jewell CM (2014). Intra-lymph node injection of biodegradable polymer particles. J Visualized Exp JoVE.

[97] Butler NS, Moebius J, Pewe LL, Traore B, Doumbo OK, Tygrett LT (2012). Therapeutic blockade of PD-L1 and LAG-3 rapidly clears established blood-stage Plasmodium infection. Nat Immunol.

[98] Suwanmanee T, Sierakowska H, Lacerra G, Svasti S, Kirby S, Walsh CE (2002). Restoration of human β-globin gene expression in murine and human IVS2-654 thalassemic erythroid cells by free uptake of antisense oligonucleotides. Mol Pharmacol.

[99] Du L, Kayali R, Bertoni C, Fike F, Hu H, Iversen PL (2011). Arginine-rich cell-penetrating peptide dramatically enhances AMO-mediated ATM aberrant splicing correction and enables delivery to brain and cerebellum. Hum Mol Genet.

[100] Ellmeier W, Jung S, Sunshine MJ, Hatam F, Xu Y, Baltimore D (2000). Severe B cell deficiency in mice lacking the Tec kinase family members Tec and Btk. J Exp Med.

[101] Yu RZ, Lemonidis KM, Graham MJ, Matson JE, Crooke RM, Tribble DL (2009). Cross-species comparison of in vivo PK/PD relationships for second-generation antisense oligonucleotides targeting apolipoprotein B-100. Biochem Pharmacol.

[102] Amantana A, Moulton HM, Cate ML, Reddy MT, Whitehead T, Hassinger JN (2007). Pharmacokinetics, biodistribution, stability and toxicity of a cell-penetrating peptide-morpholino oligomer conjugate. Bioconjug Chem.

